# Controlled Hydrothermal Synthesis and Photoluminescence of Nanocrystalline ZnGa_2_O_4_:Cr^3+^ Monospheres

**DOI:** 10.1186/s11671-017-1996-x

**Published:** 2017-03-23

**Authors:** Tian Luan, Jinhan Liu, Xiaoxue Yuan, Ji-Guang Li

**Affiliations:** 1 Key Laboratory for Anisotropy and Texture of Materials (Ministry of Education), Northeastern University, Shenyang, Liaoning 110819 China; 2 Institute of Ceramics and Powder Metallurgy, School of Materials Science and Engineering, Northeastern University, Shenyang, Liaoning 110819 China; 3 Research Center for Functional Materials, National Institute for Materials Science, 1-1 Namiki, Tsukuba, Ibaraki 305-0044 Japan

**Keywords:** ZnGa_2_O_4_, Cr red phosphor, Monospheres, Hydrothermal synthesis, Photoluminescence

## Abstract

The hydrothermal synthesis of nanocrystalline ZnGa_2_O_4_:Cr^3+^ (ZGC) red phosphor monospheres was accomplished in this work, and the effects of system pH, reactant content, reaction time, and citrate anions (Cit^3−^) on the phase and morphology evolution of the product were systematically studied. Under the optimized conditions of Cit^3−^/*M* = 1.0 molar ratio (*M* = total cations), pH = 5.0, and 0.2 mmol of Zn^2+^, well-dispersed ZGC monospheres with an average diameter of ~454 ± 56 nm (average crystallite size ~15 nm) were successfully obtained via hydrothermal reaction at 180 °C for 18 h. Cit^3+^ ions were demonstrated to be crucial to the formation of monospheres and substantially affect the pathway of phase formation. The ZGC monospheres calcined at 800 °C (average diameter ~353 ± 59 nm; average crystallite size ~30 nm) have an intensity ~6 times that of the original phosphor for the 700 nm red emission of Cr^3+^ (the ^2^E → ^4^A_2_ transition) under excitation with the O^2−^ → Ga^3+^ charge transfer band at 250 nm. Fluorescence decay analysis found that the 700 nm emission has lifetime values of ~5 ms for the ZGC phosphors.

## Background

The zinc gallate compound of ZnGa_2_O_4_ belongs to the group of cubic-structured AB_2_O_4_ normal spinels (space group: *Fd*-3*m*), in which the Zn^2+^ ions occupy the tetrahedrally coordinated A sites and the Ga^3+^ ions reside at the octahedrally coordinated B sites. The compound has been drawing increasing attention for wide applications in the fields of lighting, display, and optical imaging for biology, owing to its excellent thermal and chemical stability and wide bandgap (~4.4–4.7 eV) [1]. ZnGa_2_O_4_ is also known as a type of self-activated phosphors and may emit blue light under short UV or electron beam irradiation, owing to the occurrence of O-Ga charge transfer [1]. As a phosphor host, the Mn^2+^, Eu^3+^, and Cr^3+^ activator ions doped into the ZnGa_2_O_4_ lattice and residing at the Ga^3+^ sites are known to emit bright green, red, and red luminescence under proper excitations, respectively [2]. It is also worth noting that the transition metal ion of Cr^3+^ may emit near-infrared persistent luminescence when the chemical composition and lattice defects of ZnGa_2_O_4_ are properly manipulated, which allows the material to have potential applications in the optical imaging of vascularization, tumor, and grafted cells [3–5]. It is widely accepted that phosphor particles with a spherical shape may exhibit superior luminescence and have advantages in practical application over other morphologies, owing to the fact that the spherical shape may minimize the light scattering on particle surfaces and a denser luminescence layer can be constructed via close packing of the spheres [6, 7]. For these, developing a technique to synthesize Cr^3+^-doped ZnGa_2_O_4_ (ZnGa_2_O_4_:Cr) phosphor spheres is of practical importance. Various synthetic approaches have been established up to date for ZnGa_2_O_4_-based phosphors, typically including solid state reaction, thermal evaporation of ZnO-Ga powders, pulverizing single crystals grown by the flux method, sol-gel, electrospin, hydrothermal reaction, and chemical precipitation [8–13]. Morphology control of the product, however, yet remains an issue needed to address. We introduced in this work a hydrothermal strategy to produce well-defined ZnGa_2_O_4_:Cr^3+^ monospheres, and the effects of citrate (Cit^3−^) anions, system pH, and reactant content on the phase structure and morphology evolution were demonstrated in detail. In the following sections, we report the synthesis and photoluminescence properties of the nanostructured ZnGa_2_O_4_:Cr^3+^ monospheres.

## Methods

The stock solutions of Cr^3+^ (0.002 M) and Zn^2+^ (0.1 M) were obtained by dissolving the corresponding metal nitrates in distilled water, and the Ga^3+^ solution (0.2 M) was prepared by dissolving Ga_2_O_3_ in nitric acid (HNO_3_) via hydrothermal treatment at 100 °C. Proper amounts of the above solutions were then mixed together according to the intended chemical formula of Zn (Ga_1.995_Cr_0.005_) O_4_. Whenever needed, a certain amount of trisodium citrate (Cit^3−^) was added into the solution, followed by dilution with distilled water to a total volume of 75 mL. Under magnetic stirring, a proper amount of HNO_3_ (63 wt%) or ammonium hydroxide solution (NH_4_OH, 28 wt%) was then added to adjust the mixture to a certain pH value. After homogenizing for 30 min, the as-obtained mixture was transferred to a Teflon-lined stainless steel autoclave, which was then put into an air oven preheated to 180 °C for a certain period of hydrothermal reaction. After natural cooling to room temperature, the hydrothermal product was collected via centrifugation and washed three times with deionized water and once with ethanol, followed by drying in an air oven at 60 °C for 12 h. Calcination of the hydrothermal product was performed in the air at 800 °C for 2 h. The hydrothermal product will hereafter be referred to as *n*ZGC, where *n* is the amount of Zn^2+^ (in mmol) in the hydrothermal reaction system for the synthesis of Zn (Ga_1.995_Cr_0.005_) O_4_ phosphors.

Phase identification was made via X-ray diffractometry (XRD, Model PW3040/60, Philips, Eindhoven, The Netherlands) operated at 40 kV/40 mA, using nickel-filtered Cu-*K*α radiation (*λ* = 0.15406 nm) and a scanning rate of 5°/min in the 2*θ* range of 10°–70°. The morphology and microstructure of the products were analyzed by field emission scanning electron microscopy (FE-SEM, Model JSM-7001F, JEOL, Tokyo, Japan) under an acceleration voltage of 15 kV. Thermogravimetry of the sample was made in the air on a Model Thermo Plus TG8120 equipment (Rigaku, Tokyo), using a heating rate of 10 °C/min. Fourier transform infrared spectroscopy (FT-IR, Spectrum RXI, PerkinElmer, Shelton, CT, USA) was performed by the standard KBr method. Photoluminescence properties of the phosphors, including excitation, emission, and fluorescence decay, were measured at room temperature using an LS-55 fluorospectrophotometer (PerkinElmer).

## Results and Discussion

### Samples Synthesized Without Citrate Anions

**Fig. 1:**
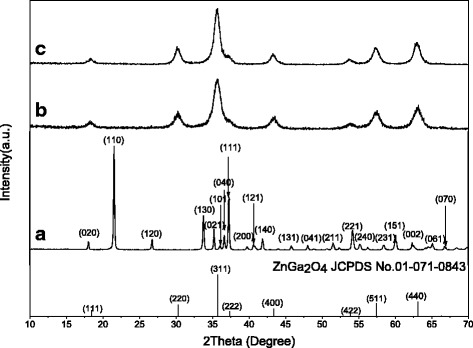
The 2ZGC products obtained by 24 h of hydrothermal reaction at 180 °C and under pH values of **a** 5, **b** 7, and **c** 9

Without the attendance of any organic molecules, the effects of system pH on the phase structure of the hydrothermal product were examined for 2 mmol of Zn^2+^ at the highest available hydrothermal temperature of 180 °C. Figure 1 shows XRD patterns of the 24 h reaction products, where it is seen that the pH = 5 sample is solely of well-crystallized α-GaOOH (JCPDS no. 06-0180) having an orthorhombic crystal structure, while those of pH = 7 and 9 can be indexed to the intended ZGC compounds (JCPDS no. 01-071-0843). This is in accordance with the literature that Ga^3+^ undergoes extensive hydration and hydrolysis in an aqueous solution to form [Ga (OH)_*x*_(H_2_O)_*y*_]^3 − *x*^ complex ion even under an acidic condition, owing to its relatively high oxidation state (3+) and rather small ionic size (0.062 nm for CN = 6) [14]. The olation reaction among [Ga (OH)_*x*_(H_2_O)_*y*_]^3 − *x*^ (removal of one water molecule via reaction of two hydroxyls) would then lead to the formation of GaOOH. The lack of any product containing Zn is primarily because the hydrolysis of Zn^2+^ ions to induce precipitation is avoided by the low solution pH of 5. This is also understandable from the view point that either ZnO or Zn (OH)_2_ is amphoteric and cannot exist under sufficiently low pH values. It can also be inferred from Fig. 1 that a higher system pH produces better crystallinity for the 2ZGC product, as seen from the sharper XRD peaks of the pH = 9 sample. Broadening analysis of the (311) diffraction with the Scherrer formula yielded average crystallite sizes of ~7 and 11 nm for the pH = 7 and pH = 9 products, respectively.

**Fig. 2:**
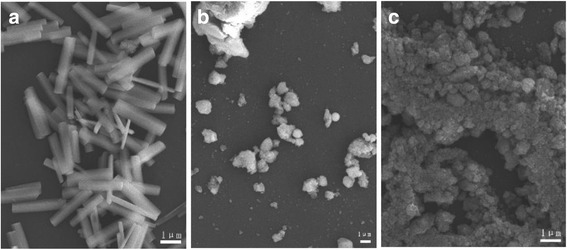
FE-SEM micrographs showing morphologies of the 2ZGC products obtained by 24 h of hydrothermal reaction at 180 °C and under pH values of **a** 5, **b** 7, and **c** 9

Figure 2 shows FE-SEM morphologies of the three products exhibited in Fig. 1. The α-GaOOH particles (Fig. 2a) are short rods with rectangular cross sections, whose lengths and diameters are up to ~3 μm and ~600 nm, respectively. Such a crystal morphology seems arising from the crystallization habit of α-GaOOH and was also observed for the products synthesized via homogeneous hydrolysis of Ga (NO)_3_ at ~90 °C [15] and via hydrothermal reaction of GaCl_3_-H_2_O-NaOH solutions at 180 °C and pH = 6–8 [16]. On the contrary, both the pH = 7 and pH = 9 products (2ZGC) are cotton- or sponge-like fluffy agglomerates, with the tiny primary crystallites unresolvable with the FE-SEM instrument.

### Optimization of the Synthesis Parameters to Yield ZGC Monospheres

**Fig. 3:**
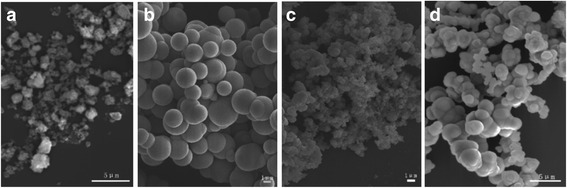
FE-SEM micrographs showing morphologies of the 2ZGC products obtained by 24 h of hydrothermal reaction at 180 °C and pH = 9. The Cit^3−^/*M* (*M* total cation) molar ratios are **a** 0.5, **b** 1, **c** 1.5, and **d** 2.0

Citrate anions (Cit^3−^) are known to be highly complexing for most of the metal cations and have been frequently used in solution-based material synthesis for reaction kinetics and morphology control. Under the same hydrothermal conditions (pH = 9 and reaction at 180 °C for 24 h), the effects of Cit^3−^ addition on particle morphology of the 2ZGC phosphors are shown in Fig. 3. It is clearly seen that spherical particles were resulted at the Cit^3−^/*M* (*M* = total cations) molar ratio *R* of 1.0, though the particles are yet not uniform in size and tend to adhere to each other (Fig. 3b). Such spherical particles were believed to have been formed via rapid simultaneous nucleation/growth in a short time duration [17, 18] and also imply the “gluing” effects of Cit^3−^ anions. At the insufficient *R* value of 0.5 (Fig. 3a), the Cit^3−^ ions were not able to well glue up the primary particles/crystallites of 2ZGC into spheres, but the observed irregularly shaped agglomerates appear denser than those shown in Fig. 1d. At the even higher *R* values of 1.5 and 2.0, the products are simultaneously composed of aggregated spheres and much smaller particles. Such a product morphology may have been resulted from substantially heterogeneous nucleation/growth, since the chelating ability of Cit^3−^ improves at a higher Cit^3−^ content, which makes the metal cations needed for 2ZGC precipitation be released in a rather slow way, and as a result, multi-step (heterogeneous) nucleation/growth would take place since no homogenization of the reaction system by stirring was performed during the hydrothermal reaction in this work [17].

**Fig. 4:**
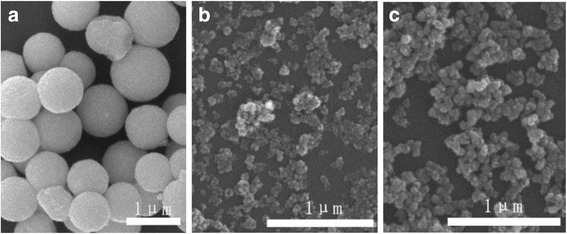
FE-SEM micrographs showing morphologies of the 0.2ZGC products obtained by 24 h of hydrothermal reaction at 180 °C. The Cit^3−^/*M* molar ratio *R* is 1.0 in each case, and the pH values are **a** 5, **b** 7, and **c** 9

To further improve the dispersion and size uniformity of the spheres shown in Fig. 3b, we lowered the Zn^2+^ content to 0.2 mmol and the effects of solution pH on particle morphology of the products (0.2ZGC) were studied at the optimal Cit^3−^/*M* molar ratio *R* of 1.0. Figure 4 shows FE-SEM morphologies of the products obtained via hydrothermal reaction at 180 °C for 24 h. It is clearly seen that lowering the Zn^2+^ content is indeed effective to produce better dispersed particles of a narrower size distribution (average size ~840 ± 160 nm) but only at the low system pH of 5 (Fig. 4a). At the higher pH values of 7 and 9, the products turned into relatively dispersed small particulates instead of spheres (Fig. 4b, c). Comparing Fig. 4a with Fig. 3b thus revealed the significant effects of cation concentration (in terms of Zn^2+^ content) on the optimal pH needed to produce spherical particles, and this can be understood as follows. Lowering the Zn^2+^ content simultaneously decreases the total amount of Cit^3−^ in solution since the *R* ratio is fixed, and this would in turn lower the gluing effects of Cit^3−^ toward the primary particles/crystallites. Under an acidic condition, for example pH = 5, the surfaces of the primary particles/crystallites are protonated, and the positive charge allows the surfaces to preferentially adsorb the negatively charged Cit^3−^ anions. As a result, the primary particles/crystallites were glued together by the adsorbed Cit^3−^ to form the spheres shown in Fig. 4a. Under the higher pH values of 7 and 9, the Cit^3−^ anions cannot be effectively adsorbed on particle/crystallite surfaces, and thus, smaller dispersed particulates were formed in the absence of sufficient Cit^3−^ gluing.

**Fig. 5:**
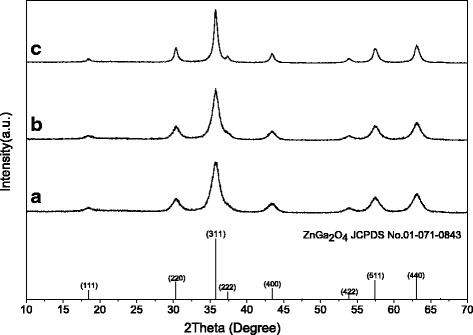
XRD patterns of the 0.2ZGC products obtained by 24 h of hydrothermal reaction at 180 °C. The Cit^3−^/*M* molar ratio *R* is 1.0 in each case, and the pH values are **a** 5, **b** 7, and **c** 9

Figure 5 shows XRD patterns of the 0.2ZGC products exhibited in Fig. 4. It is evident that they can all be well indexed to cubic-structured ZnGa_2_O_4_, whose standard diffractions were included in the figure for comparison. It is interesting to point out that the hydrothermal product synthesized in the absence of Cit^3−^ is phase-pure α-GaOOH (Fig. 1a) rather than the 0.2ZGC compound shown in Fig. 5a. This indicates that the Cit^3−^ additives have significantly modified the hydrolysis behaviors of Zn^2+^ and Ga^3+^ and altered the pathway of hydrothermal reaction, though the exact mechanism yet needs clarification. Another observation is that the sample synthesized under a lower system pH exhibited more broadened diffraction peaks, indicating that it is less well crystallized and has smaller crystallite sizes. This is understandable in view that more Cit^3−^ anions would be adsorbed on crystallite surfaces under a lower pH, which would in turn inhibit crystallite growth. Broadening analysis of the (311) diffraction with the Scherrer equation found average crystallite sizes of ~6.4, 9.7, and 10.8 nm for the products synthesized under the pH values of 5, 7, and 9, respectively.

**Fig. 6:**
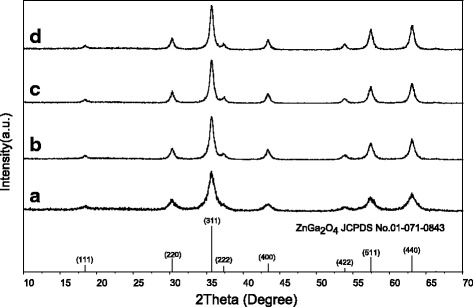
XRD patterns of the products obtained after **a** 6, **b** 12, **c** 18, and **d** 24 h of hydrothermal reaction at 180 °C. The Cit^3−^/*M* molar ratio *R* is 1.0, and the system pH is 5 in each case

Time-course phase and morphology evolution was studied for the 0.2ZGC sample under the optimized conditions of 180 °C, pH = 5, and Cit^3−^/*M* molar ratio *R* of 1.0. Figure 6 shows XRD patterns of the products obtained for different durations of hydrothermal reaction. It is seen that the 6–24-h samples are all well indexable to the ZnGa_2_O_4_ phase, with the locations and relative intensities of the diffraction peaks coincide well with the standard diffraction file (JCPDS no. 01-071-0843). It should be noted that no solid can be recovered for the shorter reaction time of 3 h. The diffraction peaks gain intensity with increasing reaction time, owing to improved crystallinity. Broadening analysis of the (311) diffraction yielded average crystallite sizes of ~8, 13, 15, and 15 nm for the ZGC phosphors obtained via 6, 12, 18, and 24 h of reaction, respectively.

**Fig. 7:**
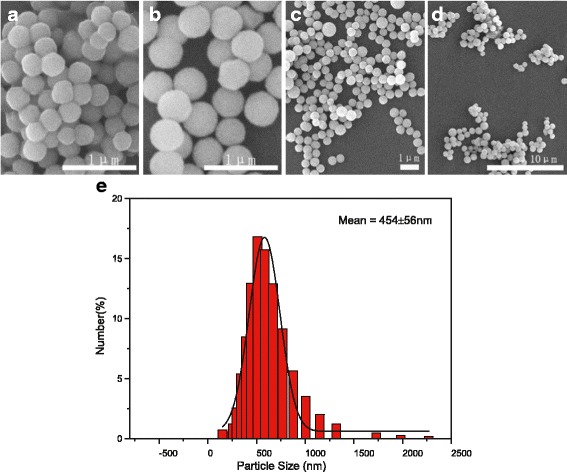
FE-SEM micrographs (**a**–**d**) showing morphologies of the 0.2ZGC products obtained after **a** 6, **b** 12, **c** 18, and **d** 24 h of reaction at 180 °C. The Cit^3−^/*M* molar ratio *R* is 1.0, and the system pH is 5 in each case. **e** is the size distribution of sample **c** obtained via dynamic laser scattering. **d** is the same sample of Fig. 4a but viewed under a lower magnification

Figure 7 shows the particle morphology of 0.2ZGC as a function of reaction time. It is seen that spherical particles have been resulted after 6 h of reaction. In view that the spheres are quite uniform in shape and size (~295 ± 34 nm) while 3 h of reaction did not yield any solid, it can thus be inferred that the spheres were formed in a rather short duration of time via rapid simultaneous nucleation/growth as aforementioned. The average size of the spheres increases with increasing reaction time, which reached ~422 ± 47 nm at 12 h, ~454 ± 56 nm at 18 h, and ~840 ± 158 nm at 24 h. The size increment is largely caused by Ostwald ripening, which is enhanced by the acidic reaction condition (pH = 5). It is also seen that the 18 h product has smoother particle surfaces and a more spherical shape than the 6 and 12 h products and is better dispersed and more uniform in particle size than the 24 h product. Indeed, particle sizing via laser diffraction found that the 18 h product exhibits an almost single modal size distribution (Fig. 7e) and has an average diameter of ~454 ± 56 nm. This sample was therefore chosen for further characterizations.

**Fig. 8:**
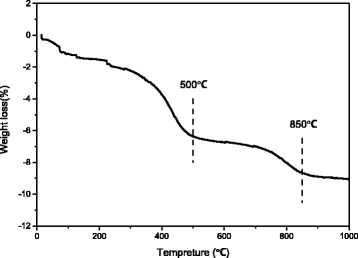
TG trace for the 18 h product shown in Fig. 7c

TG analysis of the 18 h product found three major stages of weight losses and a total weight loss of ~9 wt% up to 1000 °C (Fig. 8), the origin of which will later be clarified with the results of FT-IR. It is clear that the weight loss of the sample has almost terminated at ~800 °C.

**Fig. 9:**
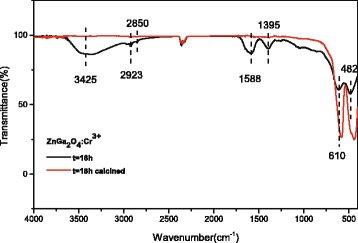
FT-IR spectra for the 18 h product (*black line*) and that calcined at 800 °C for 2 h (*red line*)

FT-IR spectroscopy of the as-synthesized 18 h product found O–H stretching vibration of water molecules at ~3425 cm^−1^, COO– absorptions of Cit^3−^ at ~1588 and 1395 cm^−1^, and –CH_2_– vibrations at ~2923 and 2850 cm^−1^ [19–21]. It is noteworthy that the O–H bending mode of water, usually occurring at ~1640 cm^−1^, overlaps with the ~1588 cm^−1^ vibration of COO– and contributes to the broadening of the band in the ~1440–1750 cm^−1^ region (the black line). The two bands located at ~610 and 482 cm^−1^ can be ascribed to Zn–O and Ga–O vibrations, respectively [22]. After 800 °C calcination, the absorptions corresponding to H_2_O and COO– groups are barely observable while metal-oxygen vibrations were enhanced due to increased crystallinity of the sample (the red line) (Fig. 9). In addition, the twin bands at ~2300 cm^−1^ observed for both the original and calcined powders are arising from atmospheric CO_2_. The FT-IR results thus suggest that the weight loss observed for the original 18 h sample in Fig. 8 is largely due to dehydration and the removal of adsorbed Cit^3−^ anions.

**Fig. 10:**
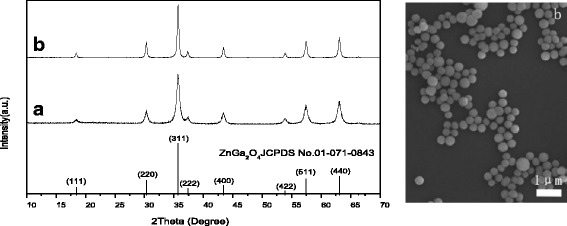
A comparison of the 18 h sample before (*line a*) and after (*line b*) calcination at 800 °C (*left panel*) and FE-SEM particle morphology of the calcination product (*right panel*)

The left-hand panel of Fig. 10 compares XRD patterns of the 18 h powder before and after calcination at 800 °C. It is seen that the calcination did not alter the phase purity but substantially improved the crystallinity of the phosphor. Analysis with the (311) diffraction found average crystallite sizes of ~15.0 and 30.4 nm and lattice constants of ~0.83402 and 0.83375 nm for the as-synthesized and calcined powders, respectively. The lattice parameters assayed in this work are close to the value of *a* = 0.83349 nm for ZnGa_2_O_4_ in the standard data file. FE-SEM observation indicated that the calcination product is solely composed of dispersed monospheres, but the average particle size contracted from ~454 ± 56 to 353 ± 59 nm due to the mass loss and densification during calcination.

**Fig. 11:**
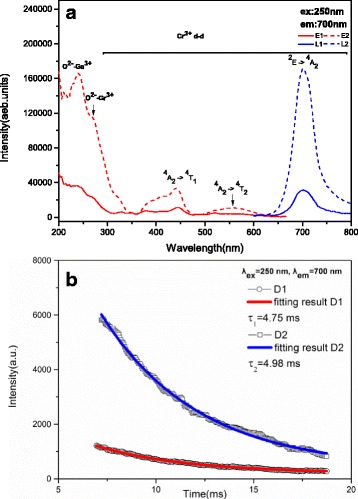
Photoluminescence excitation (*lines E1* and *E2*) and emission (*lines L1* and *L2*) spectra (**a**) and fluorescence decay kinetics (**b**) for the as-synthesized 18 h sample (*lines E1*, *L1*, and *D1*) and that calcination at 800 °C for 2 h (*lines E2*, *L2*, and *D2*)

Figure 11a shows the excitation and emission spectra of the as-synthesized and 800 °C calcined 0.2ZGC phosphors (the 16 h product). It can be seen that the excitation spectrum obtained by monitoring the ~700 nm red emission of Cr^3+^ is composed of four main bands covering a wide spectral region from ultraviolet to red, with those centered at ~250, 275, 440, and 550 nm arising from O^2−^ → Ga^3+^ charge transfer, O^2−^ → Cr^3+^ charge transfer, the ^4^A_2_ → ^4^T_1_ d-d transition of the Cr^3+^ activator, and the ^4^A_2_ → ^4^T_2_ d-d transition of Cr^3+^, respectively [23]. The appearance of O^2−^ → Ga^3+^ charge transfer band by monitoring Cr^3+^ emission implies the occurrence of efficient Ga^3+^ → Cr^3+^ energy transfer. Calcination at 800 °C greatly improves the excitation intensity, owing to the removal of water molecules, organic residues, and particularly the improved crystallinity of the phosphor powder. Exciting the phosphor with the O^2−^ → Ga^3+^ charge transfer band at 250 nm produced the ^2^E → ^4^A_2_ emission of the Cr^3+^ activators at ~700 nm [1], which further confirms the occurrence of Ga^3+^ → Cr^3+^ energy transfer. It is seen from the PL spectra that the phosphor calcined at 800 °C has an emission intensity ~6 times that of the as-synthesized one. Fluorescence decay kinetics of the 700 nm emission under 250-nm excitation is shown in Fig. 11b. Both of the decay curves can be well fitted to the single exponential function of *I* = *I*_0_ exp (−*t*/*τ*), from which the lifetime of the 0.2ZGC phosphor was calculated to be 4.75 ± 0.07 ms for the as-synthesized sample and 4.98 ± 0.06 ms for the calcined sample. The lifetime determined herein is a little longer than the reported values of ~1.4–2.5 ms but is on the same order of magnitude [23].

## Conclusions

Nanocrystalline ZnGa_2_O_4_:Cr^3+^ (ZGC) monospheres were synthesized in this work via hydrothermal reaction at 180 °C and in the presence of Cit^3−^ ions, which emit red emission at 700 nm (the ^2^E → ^4^A_2_ transition of Cr^3+^) upon short UV excitation with the O^2−^ → Ga^3+^ charge transfer band at 250 nm. The optimal processing parameters were determined to be Cit^3−^/M = 1.0 molar ratio (*M* = total cations), pH = 5.0, 0.2 mmol of Zn^2+^, and a reaction time of 18 h. Calcining the as-synthesized ZGC monospheres at 800 °C for 2 h brought about an ~6-fold intensity increment for the 700 nm emission, owing to dehydration, removal of organic residues, and crystallinity improvement. The phosphor monospheres were analyzed to have lifetime values of ~5 ms for the 700 nm red emission.
